# Brain Tumor Resection in Elderly Patients: Potential Factors of Postoperative Worsening in a Predictive Outcome Model

**DOI:** 10.3390/cancers13102320

**Published:** 2021-05-12

**Authors:** Paolo Ferroli, Ignazio Gaspare Vetrano, Silvia Schiavolin, Francesco Acerbi, Costanza Maria Zattra, Marco Schiariti, Matilde Leonardi, Morgan Broggi

**Affiliations:** 1 Department of Neurosurgery, Fondazione IRCCS Istituto Neurologico Carlo Besta, 20133 Milan, Italy; paolo.ferroli@istituto-besta.it (P.F.); francesco.acerbi@istituto-besta.it (F.A.); costanzamaria.zattra01@universitadipavia.it (C.M.Z.); marco.schiariti@istituto-besta.it (M.S.); morgan.broggi@istituto-besta.it (M.B.); 2 Neurology, Public Health and Disability Unit, Fondazione IRCSS Istituto Neurologico Carlo Besta, 20133 Milan, Italy; silvia.schiavolin@istituto-besta.it (S.S.); matilde.leonardi@istituto-besta.it (M.L.)

**Keywords:** brain tumors, complications, craniotomy, elderly, KPS, mortality, predicting factors, outcome

## Abstract

**Simple Summary:**

Brain tumor surgery in older patients is becoming more relevant, considering that the proportion of older adults being treated for cancer is rising, whereas some pivotal studies in neuro-oncology comprised young patients only. The knowledge of possible predictors of outcome should be included in the preoperative assessment to make the best possible decision in terms of management. We present a case series of 143 patients older than 65 years, intending to identify the possible factors predicting the risk of clinical worsening after elective surgical resection of intracranial tumors in elderly patients. We found that postoperative complications occurrence and preoperative surgical complexity significantly influence the outcome in this subgroup of patients, whereas postoperative complications were the only factor with an impact also at long-term follow-up.

**Abstract:**

The decision of whether to operate on elderly patients with brain tumors is complex, and influenced by pathology-related and patient-specific factors. This retrospective cohort study, based on a prospectively collected surgical database, aims at identifying possible factors predicting clinical worsening after elective neuro-oncological surgery in elderly patients. Therefore, all patients ≥65 years old who underwent BT resection at a tertiary referral center between 01/2018 and 12/2019 were included. Age, smoking, previous radiotherapy, hypertension, preoperative functional status, complications occurrence, surgical complexity and the presence of comorbidities were prospectively collected and analyzed at discharge and the 3-month follow-up. The series included 143 patients (mean 71 years, range 65–86). Sixty-five patients (46%) had at least one neurosurgical complication, whereas 48/65 (74%) complications did not require invasive treatment. Forty-two patients (29.4%) worsened at discharge; these patients had a greater number of complications compared to patients with unchanged/improved performance status. A persistent worsening at three months of follow-up was noted in 20.3% of patients; again, this subgroup presented more complications than patients who remained equal or improved. Therefore, postoperative complications and surgical complexity seem to influence significantly the early outcome in elderly patients undergoing brain tumor surgery. In contrast, postoperative complications alone are the only factor with an impact on the 3-month follow-up.

## 1. Introduction

In the past decades, the world population has progressively aged, especially in Western countries. Increased life expectancy, due to improved socio-economic and health conditions, is associated with a growing surge in health demands [1]. In 2018, nearly one-fifth of the population in the European Union (EU) was aged 65 years old (y.o.) or older [2], with Italy’s 35.2% being the highest value in the EU [3,4]. This aspect is counterbalanced by the progressive extension of the working age, up to 70 years of age. However, elderly patients present more comorbidities and are potentially more susceptible to surgical complications leading to dependency and long-term care. Furthermore, the incidence of many neurosurgical diseases, including brain tumors (BT), increases in elderly patients [5,6]. These trends imply a rise in the neurosurgical treatment demand within the elderly population [7,8].

Whether to operate on elderly patients with BT is sometimes a difficult decision, which is influenced by several patient-specific factors, including preoperative functional status and pathology-related aspects, such as presumed histology and preoperative surgical complexity [8,9,10]. Other factors to consider are life expectancy and quality of life; amidst all this complexity, the knowledge of possible predictors of outcome should be included in the preoperative assessment to make the best possible decision in terms of management.

Recently, several studies about the safety of neurosurgical procedures in the elderly have been presented, but most of the existing literature on this issue measures surgical outcome only in terms of mortality, perioperative complications, and early postoperative outcome [5,8,9,11,12]. Moreover, some pivotal studies in neuro-oncology comprised patients younger than 70 y.o., excluding older ones [13]. On the other hand, the role of predicting factors on outcome in terms of functional status has rarely been considered and assessed, especially in neuro-oncological surgery. This study aims to explore the existence and the role of risk factors of early (at hospital discharge) and late (at three months) postoperative clinical worsening in patients older than 65 y.o. undergoing surgical resection of intracranial tumors.

## 2. Materials and Methods

A monocentric, prospectively collected database, dedicated to reporting patients’ clinical and radiological data, was retrospectively reviewed. We included in the study all consecutive patients ≥65 y.o. and harboring a BT who were surgically treated between 1 January 2018 and 31 December 2019 in the Neurosurgical Department (II Division) of a high-volume, tertiary, national referral center (Fondazione IRCCS Istituto Neurologico Carlo Besta in Milan, Italy). The database was approved by the Institutional Ethics Committee. Only elective surgeries aimed at so-called “maximal safe tumor resection” were considered, thus excluding needle and open biopsies. The Karnofsky Performance Scale (KPS) [14], the more widely variable used in oncology practice with a very high reliability [15], was used to classify each patient’s health status before surgery, at discharge, and at a 3-month follow-up (FU). The Charlson Comorbidity Index (CCI) was used to classify comorbidities: it includes 17 clinical conditions which are reported as secondary diagnoses; each of them is assigned a number ranging from 1 to 6, and the final score, which is also weighed on age, corresponds to the sum of all the items [16]. Other conditions not included in the CCI, such as smoking, previous radiotherapy, hypertension, body mass index (BMI), and postoperative complications, were selectively analyzed to determine their relevance and possible role in clinical worsening.

Finally, surgical complexity was also analyzed: it was prospectively graded according to the Milan Complexity Scale (MCS) [10]. The MCS is composed of five preoperative parameters: involvement of major brain vessels, surgery in eloquent areas, posterior fossa location, involvement of cranial nerves, and tumor size larger than 4 cm (Table 1).

The resulting score ranges from 0 to 8: higher scores correspond to greater case complexity and a higher chance of clinical worsening after surgery [10]. Complications were classified as any deviation from an uneventful postoperative course occurring within 30 days from surgery [16], and they were recorded based on the required treatment, according to the Landriel classification (Appendix A, Table A1) [17,18].

### 2.1. Surgical Protocol

All patients received a standard preoperative evaluation, which included blood testing and cardiologic, anesthesiologic, and neurologic assessment. All patients judged suitable for surgery and general anesthesia underwent a preoperative Magnetic Resonance (MR) examination, including volumetric sequences after intravenous (i.v.) paramagnetic contrast agent administration for neuronavigation during tumor removal. When indicated, some of the most advanced technologies and tools, such as intraoperative ultrasound and contrast-enhanced ultrasound [19], photodynamic detection with indocyanine-green and sodium fluorescein (5 mg/kg) [20,21,22], and intraoperative neurophysiological monitoring [23], were employed, according to the institutional and standard international protocols. Intensive Care Unit (ICU) admission for postoperative monitoring was decided together by the operating neurosurgeon and the anesthesiologist, based on the complexity of the surgery, the patient’s general clinical status, and tumor location: for example, all patients undergoing posterior fossa removal were admitted to the ICU.

All patients underwent postoperative computed tomography (CT) scan within 24 h after surgery to exclude acute complications and MR with i.v. contrast agent administration within 72 h to evaluate the extent of tumor resection. Tumors histological classification was based on the 2016 WHO classification of Central Nervous System tumors [24].

### 2.2. Statistical Analysis

Descriptive statistics were used to show the main sample features, specifically numbers and percentages for categorical variables and means ± standard deviation for quantitative variables. No statistical power calculation was conducted prior to the study and the sample size was based on the available data. There were no missing data concerning the variables included in the study, while the outliers were evaluated but no action was necessary. The comparisons between preoperative KPS, KPS at discharge, and three-month FU were performed using Friedman’s analysis of variance with a Wilcoxon post-hoc test, as q-q plots showed that data were not normally distributed. A two-tailed significance level of α = 0.016 was adopted because of Bonferroni’s adjustment to reduce the type 1 error due to the multiple comparisons.

Patients were dichotomized into improved/unchanged and worsened based on the difference between preoperative KPS and KPS at discharge and between preoperative KPS and KPS at the three-month FU. The worsened group was composed of patients with a 10-point or greater decrease in KPS at discharge and at three months compared with preoperative KPS. While there is no established minimal clinically important difference for KPS after intracranial tumor surgery, we and other groups defined as “postoperative functional impairment” a 10-point or greater decrease in KPS [10]; this value was deliberately chosen as opposed to a dynamic cutoff with different steps depending on baseline status, in order not to overlook subtle differences in performance, since even minor decreases in performance as judged by clinical scales can be perceived as devastating by patients.

Age, smoking, previous radiotherapy, hypertension, preoperative KPS, MCS score, comorbidities, BMI, and complications were compared between the improved/unchanged and worsened groups at discharge and FU using chi-squared or Fisher test for categorical variables and Mann–Whitney *U* test for quantitative variables because data were not normally distributed. The two-tailed significance level was set at α = 0.006 because of the multiple comparisons. Furthermore, effect size (ES) was calculated using G*Power as an indicator of the difference between improved/unchanged and worsened groups with respect to these variables. Values of 0.2 to 0.3 were considered as a small ES, around 0.5 as medium and from 0.8 to infinity as large.

Patients with complication were compared to patients without complication with respect to previous radiotherapy, MCS, smoking, primary vs secondary surgery, and benign vs malignant tumors and using *t* test for MCS and chi-squared or Fisher test for categorical variables. The two-tailed significance level was set at α = 0.01 because of the multiple comparisons.

Univariate logistic regression analysis was used to investigate the relationship between each of the previous variables (age, smoking, previous radiotherapy, hypertension, preoperative KPS, MCS score, comorbidities, BMI, complications) with the two clinical outcomes (improved/unchanged versus worsened at discharge and FU). Variables with a *p*-value ≤ 0.05 were further analyzed in combination with multivariate logistic regressions. Comparisons between improved/unchanged and worsened patients and logistic regression analysis were also performed separately in the groups 65–74 y.o. and ≥75 y.o. Finally, complications and KPS change at discharge and FU were compared between patients aged 65–74 and patients older than 75 using the chi-square test for complication and the Mann–Whitney *U* test for the change in KPS scores. All the statistical analyses were preplanned specifically for this study, and were conducted using SPSS 24.0 statistical software.

## 3. Results

### 3.1. Patients Characteristics

A total of 143 patients, 78 (55%) of whom were female, were included in the analyses. The mean age was 71 y.o. (sd = 4.6; range 65–86). The most frequent tumor types were meningiomas (49, 34.3%) and glioblastomas (GBM) (41, 28.7%). Table 2 reports the main clinical characteristics, including mean KPS scores (preoperative, at discharge, and FU) and their longitudinal change.

### 3.2. Surgical Complications

Among all patients, 65 (46%) had at least one neurosurgical complication, according to the Landriel scale [25]. They were distributed as follows: 48 (33.6%) grade I complications, which did not require invasive treatment (examples include but are not confined to superficial wound infection, seizures, and extra central nervous system infections treated with antibiotics); 9 (6.3%) grade II complications, which required invasive treatment (such as re-operations due to surgical hematoma or cerebrospinal fluid leak repair; wound infection requiring surgical debridement; or shunt placement for development of hydrocephalus); and 4 (2.8%) grade III complications, which required admission and prolonged care in the ICU. The early (within one month from surgery) mortality rate (Landriel grade 4) was 2.8%, and specifically, the following patients died: one 68 y.o. patient with a recurrent huge skull base atypical meningioma, who died from uncontrolled brain edema; one 72 y.o. male affected by a right temporal metastasis, who died from postoperative intracranial hemorrhage; one 79 y.o. male with multiple brain metastasis, who died following massive ischemic stroke; and one 77 y.o. female harboring a recurrent bulbar hemangioblastoma, who died from complications related to postoperative hematoma development.

No significant differences were found between patients with complications and patients without complication with respect to previous radiotherapy, MCS, smoking, primary vs secondary surgery, and benign vs malignant tumor.

### 3.3. Functional Outcome

KPS scores significantly change over the time point evaluations (*ϰ^2^* (2) = 10.987; *p* = 0.004). Wilcoxon tests were used to follow up this finding: no statistically significant differences were found, after a median follow-up of 110 days, between mean preoperative KPS and mean KPS at FU (*Z* = −0.485; *p* = 0.628); the mean KPS score decreased at discharge (*Z* = −2.550; *p* = 0.011), but it improved from discharge to FU (*Z* = −3.328; *p*= 0.001). In total, 101 (70.6%) patients remained stable or improved at discharge and 42 (29.4%) worsened; the latter had a higher number of complications (*p* = 0.000) and a higher MCS score (*p* = 0.005) compared to patients with unchanged or improved KPS (Table 3).

A persistent worsening at the three-month FU was noted in 29 (20.3%) patients; again, this subgroup presented more complications (*p* = 0.000) than patients who remained equal or improved (Table 4). The same results were found when comparing improved/unchanged and worsened patients in the 65–74 y.o. and older than 75 y.o. groups, separately. The only exception was found in the group ≥75 y.o., in which patients worsened at FU had a higher BMI, and those worsened at discharge had an MCS score similar to improved/unchanged patients.

### 3.4. Univariate and Multivariate Analysis

The results of univariate logistic regression showed that the MCS complexity score was associated with an increased odd of worsening at discharge (β = 0.219; Wald = 5.929; OR = 1.244; *p* = 0.015); more specifically, for each extra point of MCS score, the odds of worsening increased by 1.244 times. However, the more relevant factor associated with a short-term worsening (evaluated at discharge) was the occurrence of any complication (β = 3.260; Wald = 32.504; OR = 26.037; *p* = 0.000). Moreover, the presence of a complication determined an increased odd of remaining worsened at the three-month FU as well (β = 2.146; Wald = 16.454; OR = 8.546; *p* = 0.000). The multivariate analysis of MCS score and complications combined revealed that only complication development was a significant predictor of clinical worsening at discharge.

The univariate logistic regression analysis did not demonstrate a meaningful relationship between age and long-term functional status worsening. Additionally, the other analyzed factors, such as smoking, previous radiotherapy, hypertension, and BMI, were not associated with functional status impairment. Not even the presence of comorbidities showed a relationship with KPS score at hospital discharge or three-month FU. Similar results were found in multivariate analysis performing logistic regressions separately in the 65–74 y.o. and older than 75 years groups.

## 4. Discussion

The present study confirms that surgical resection of BT in patients older than 65 is feasible and rather safe [5,7,26]. The recently growing interest in elderly neuro-oncological patients has generated a series of studies that highlight how increased age (≥65 years), alone does not represent an independent risk factor associated with worse outcomes [27]. It has been demonstrated that frailty, described as the “clinically recognizable state of increased vulnerability resulting from age-associated physiological decline” [27], is per se an outcome predictor, but not the only one. Therefore, other factors need to be stratified in a predictive model. Due to cranial neurosurgery complexity at any age, mortality rates are one of the most frequently assessed parameters [28,29]. Notwithstanding, as recently pointed out by Schar et al. [26], other operative risk-stratification models are needed to try to predict the outcome following neurosurgical procedures. Such models should analyze all factors possibly associated with intra- and postoperative complications. Therefore, dedicated prospectively-collected registries comprising all preoperative data (demographic-, patient-, and pathology-related) along with postoperative data, especially complications and functional outcomes, are warranted to identify possible risk factors of worsening after surgery. In the present study, with the use of such a prospectively-collected institutional registry, we analyzed the impact of several items on the outcome of BT surgery in elderly patients.

### 4.1. Complications and Other Possible Predicting Factors

In the present series, the most relevant factor associated with short-term (at discharge) clinical worsening was the occurrence of postoperative complications, no matter which grade they were. The presence of a complication determined an increased likelihood of remaining worsened at the three-month FU as well. We report a complication rate of 46%, which might seem fairly high; yet, it should be noted that our definition of complication implies “any deviation from a fully uneventful postoperative course” [16,25]. Using a similar definition, the present complication rate is slightly higher than that found in a previous series of BT patients of any age [10]; notably, most of these complications were Landriel grade 1 (74%) and did not require invasive treatment. These data confirm those of Maldaner et al. [7], in particular, a higher rate of perioperative complications in elderly patients. Shahrestani et al. recently highlighted that the complication rate significantly differs between frail and non-frail elderly patients immediately following cranial surgery [27]. In contrast, they also suggest that there may be no significant difference in long-term follow-up. Other prognostic factors, such as smoking and comorbidities, are often associated with complications and postoperative worsening among older adults undergoing any type of elective surgery [30]. Interestingly, in our series, most of these factors, including previous radiotherapy and hypertension, did not appear as significant predictors of clinical worsening.

Moreover, it seems that the role of tumor type is not a major determinant in predicting outcome: frailty in geriatric neurosurgical patients has an impact on outcome regardless of histology, also in large patients’ cohorts [27]. The abovementioned considerations are not new to recent literature: Ius et al., in a multicentric series of elderly GMB patients, excluded any correlation between the presence of comorbidities (evaluated via the CCI) and outcome or overall survival [9]. The possible explanation is represented by an intuitive “selection bias”: elderly patients with high KPS scores and stabilized comorbidities are proposed to undergo major surgery, whereas patients with diffuse or multicentric BT or relevant morbidities are usually submitted to palliative care or minor surgical procedures as needle biopsy. Therefore, these patients are excluded from further analysis, as happened in our series. However, one-third of the postoperative KPS decline was reversible (i.e., improved between discharge and follow-up) and 74% of complications were low-grade. Furthermore, the preoperative complexity was fairly high with 36.4% of tumors treated being located in an eloquent area; these were all operated with the aid of a combination of functional MR, tractography, intraoperative monitoring, and, in selected cases, awake surgery that allowed us to push the resection very close to the eloquent tissue, which often implies a temporary worsening of neurological functions. Indeed, we explain our data by the high preoperative complexity that is rather typical of a tertiary referral center which accepts challenging cases previously refused by peripheral hospitals.

### 4.2. The Impact of Surgical Complexity on Outcome

The other significant predictor of worsening at discharge was preoperative case complexity, evaluated through the MCS; this means that the higher the MCS score, the higher the risk of postoperative deterioration. These findings further confirm what we found when we developed the scale [10]. Although the items included in the MCS (involvement of major vessels and cranial nerves, eloquent areas surgery, posterior fossa location, tumor size larger than 4 cm) are rather intuitive, the literature lacks prospective studies evaluating predictive models of complexity and their impact on surgical outcome. Applying such models to specific subgroups of patients that are intrinsically vulnerable to surgery (e.g., over 65 y.o.) [1,5,27] may be helpful not only to predict the postoperative course but also to make the safest decision in terms of who receives indication for BT surgery. In our study, the MCS score was associated with an increased odd of neurological worsening at discharge. If the data analysis considers only the early postoperative course, usually evaluated at the 30 days FU [7,26,31], surgical complexity significantly impacts the outcome. However, when considering the long-term outcome, this impact decreases: in our series, at multivariate analysis, the complexity score was no longer statistically significant at the three-month FU.

A possible explanation of this effect is that some of the early postoperative new deficits often appear to be transient. Many patients were able to recover or even improve their condition, either spontaneously or through physical therapy. This supports what Schar et al. recently showed [26], namely that elderly neurosurgical patients pose a challenge for the immediate postoperative management, including a prolonged hospital length of stay, need for intensive care, and increased costs. These patients also often require transfer to rehabilitation hospitals or prolonged physical therapy, which can eventually lead to functional recovery in many cases.

### 4.3. Does Age Matter?

The relationship between surgical complexity and long-term outcome (both in the univariate and multivariate analysis) is also confirmed when considering patients aged 65–74 y.o. and patients older than 75 y.o. separately. These results indicate that, once again, age alone should not be considered as a limit nor as an absolute contraindication to BT surgery. The percentage of worsened patients is similar to that found in a previous series of 746 operated BT in patients of any age [10]. The fact that older age does not appear a determining factor in shaping the postoperative outcome is recently emerging as a common finding among different centers, as Schwartz, Schar, and others pointed out [9,11,26,27,31,32,33,34]. On the other hand, the occurrence of complications of any grade and type (surgical and non-surgical) in the present elderly population seems to be slightly higher (46 vs. 41%) when compared to other series more heterogeneous in terms of age [10], and this has a significant impact on outcome both at discharge and at the three-month FU, regardless of the complication grade [17,25]. In other words, probably due to the intrinsic frailty of the elderly [1,27], they are less fit to face a complication, whether it is a minor one or, even more so, when it requires a new surgical intervention.

### 4.4. Limitations

Our study’s limitations must be considered: first and foremost, this is a retrospective single tertiary referral center study. Therefore, our results may not be generalizable to the broader neurosurgical community due to differences, for example, in perioperative care, technical resources, and patient discharge patterns, which could affect the post-operative course and the onset of complications. Furthermore, we acknowledge a selection bias in our case series since it consists of a group of patients who were considered fit enough for surgery by the neurosurgeon and the anesthesiologist. This may hamper the extension of our findings to all patients aged ≥65 years. Additionally, similarly to other studies [9,27], patients with diffuse or multicentric BT, submitted to palliative care or minor surgical procedures, were excluded from further analysis. Another potential weakness to consider is the variety of tumor types lumped together in this analysis. However, the early outcome between meningiomas, gliomas, metastases, and the many other tumor types included in this study should not vary substantially; other groups support our considerations of the limited role of tumor type in the early outcome of the specific subset of patients included in our series [27]; conversely, it is clear that our study population does not allow us to make long-term consideration (e.g., survival time, progression-free survival, etc.) since in the long term, tumor biology plays a crucial role. Finally, the sample size could have affected our results on significant differences among groups and longitudinal differences. However, our results are rather promising since they were also confirmed by considering patients between 65 and 74 y.o. and patients older than 75 y.o., separately. Further multicenter studies on larger samples may confirm these findings.

## 5. Conclusions

In the present study, postoperative complications and surgical complexity significantly influenced the risk of clinical worsening after BT surgery in patients >65 years old. In other words, due to their intrinsic frailty, elderly patients are less fit to face a complication, whether it is a minor one or, even more so, when it requires a new surgical intervention.

According to our results, age, smoking, comorbidities, and previous radiotherapy should not be considered as primary factors in outcome prediction models applied to elderly patients harboring BT. The only element with a strong correlation to postoperative worsening at the three-month FU was the occurrence of complications.

## Figures and Tables

**Table 1:** The Milan Complexity Scale (MCS) grading.

Variable	Score
Major brain vessel manipulation	
No	0
Yes	1
Posterior fossa	
No	0
Yes	1
Cranial nerve manipulation	
No	0
Yes	2
Eloquent area	
No	0
Yes	3
Tumor size	
0–4 cm	0
≥4.1 cm	1
Total score	0–8

**Table 2:** Patients’ characteristic and clinical outcomes.

Mean Age	71 ± 4.6
65–74 y.o.	109 (76.2%)
≥75 y.o.	34 (23.8%)
Diagnosis	
Glioblastomas	41 (28.7%)
Meningiomas	49 (34.3%)
Adenomas	15 (10.5%)
Metastases	12 (8.4%)
Chordomas	4 (2.8%)
Craniopharyngiomas	3 (2.1%)
Low-grade gliomas	2 (1.4%)
Anaplastic astrocytomas	2 (1.4%)
Neurinomas	2 (1.4%)
Epidermoid cysts	2 (1.4%)
Other	11 (7.7%)
Tumor classification	
Intra-axial	64 (44.8%)
Extra-axial	79 (55.2%)
Infratentorial	19 (13.3%)
Supratentorial	124 (86.7%)
Side	
Left	61 (42.7%)
Right	55 (38.5%)
Bilateral/midline	27 (18.9%)
KPS scores (median; range)	
Preoperative KPS	90; 30–100
KPS at discharge	80; 0–100
KPS at follow-up	90; 0–100
Complications (Landriel classification)	
No complications	78 (54.5%)
Grade I	48 (73.8%)
Grade II	9 (13.8%)
Grade III	4 (6.2%)
Grade IV	4 (6.2%)

**Table 3:** Comparison between worsened and improved/unchanged patients at discharge (number and percentage for categorical variables and mean ± standard deviation for quantitative).

Clinical Variables	Worsened at Discharge	Improved or Unchanged at Discharge	*p*-Value	Effect Size
Age	70.7 ± 4.2	71.2 ± 4.8	0.739	0.10
Preoperative KPS (median; range)	90; 60–100	90; 30–100	0.762	0.11
MCS	3.3 ± 2.0	2.3 ± 2.1	0.005 *	0.47
CCI	5.1 ± 2.1	5.1 ± 1.4	0.440	0.02
BMI	25.7 ± 3.9	26.3 ± 4.2	0.488	0.16
Smoke	-	-	0.693	0.07
Yes	6 (33.3%)	12 (66.7%)	-	-
No	36 (28.8%)	89 (71.2%)	-	-
Previous radiotherapy	-	-	0.606	0.09
Yes	6 (25%)	18 (75%)	-	-
No	36 (30.3%)	83 (69.7%)	-	-
Hypertension	-	-	0.580	0.10
Yes	22 (27.5%)	58 (72.5%)	-	-
No	20 (31.7%)	43 (68.3%)	-	-
Complications	-	-	0.000 *	1.44
Yes	38 (58.5%)	27 (41.5%)	-	-
No	4 (5.1%)	74 (94.9%)	-	-

* *p* ≤ 0.006; KPS = Karnofsky Performance Scale; MCS = Milan Complexity Scale; CCI = Charlson Comorbidity Index; BMI = Body Mass Index.

**Table 4:** Comparison between worsened and improved/unchanged patients at follow-up (number and percentage for categorical variables and mean ± standard deviation for quantitative).

Clinical Variables	Worsened at Follow-Up	Improved or Unchanged at Follow-Up	*p*-Value	Effect Size
Age	71.2 ± 4.6	71 ± 4.6	0.793	0.05
Preoperative KPS (median; range)	90; 50–100	90; 30–100	0.550	0.16
MCS	3 ± 1.7	2.5 ± 2.2	0.072	0.25
CCI	5.1 ± 2.1	5.1 ± 1.5	0.836	0.05
BMI	26.0 ± 3.9	26.1 ± 4.2	0.968	0.03
Smoke	-	-	0.024	na
Yes	0 (0%)	18 (100%)	-	-
No	29 (23.2%)	96 (76.8%)	-	-
Previous radiotherapy	-	-	1.000	0.01
Yes	5 (20.8%)	19 (79.2%)	-	-
No	24 (20.2%)	95 (79.8%)	-	-
Hypertension	-	-	0.177	0.28
Yes	13 (16.3%)	67 (83.8%)	-	-
No	16 (25.4%)	47 (74.6%)	-	-
Complication	-	-	0.000 *	1.24
Yes	24 (36.9%)	41 (63.1%)	-	-
No	5 (6.4%)	73 (93.6%)	-	-

* *p* ≤ 0.006; KPS = Karnofsky Performance Scale; MCS = Milan Complexity Scale; CCI = Charlson Comorbidity Index; BMI = Body Mass Index; na = not applicable.

## Data Availability

The data presented here are available on request from the corresponding author.

## Appendix A

**Table A1:** Classification of Neurosurgical Complications according to Landriel [17] with permission from Elsevier.

Grade	Description
Grade I	Any non-life-threatening deviation from normal postoperative course, not requiring invasive treatment
Grade Ia	Complication requiring no drug treatment
Grade Ib	Complication requiring drug treatment
Grade II	Complication requiring invasive treatment such as surgical, endoscopic, or endovascular interventions
Grade IIa	Complication requiring intervention without general anesthesia
Grade IIb	Complication requiring intervention with general anesthesia
Grade III	Life-threatening complications requiring management in the ICU
Grade IIIa	Complication involving single organ failure
Grade IIIb	Complication involving multiple organ failure
Grade IV	Complication resulting in death
Surgical Complications	Adverse events that are directly related to surgery or surgical technique
Medical Complications	Adverse events that are not directly related to surgery or surgical technique
